# Long-Term Sertraline Intake Reverses the Behavioral Changes Induced by Prenatal Stress in Rats in a Sex-Dependent Way

**DOI:** 10.3389/fnbeh.2017.00099

**Published:** 2017-05-29

**Authors:** Inês Pereira-Figueiredo, Orlando Castellano, Adelaida S. Riolobos, Graça Ferreira-Dias, Dolores E. López, Consuelo Sancho

**Affiliations:** ^1^Neuroscience Institute of Castilla y León, Institute for Biomedical Research of Salamanca (IBSAL), University of SalamancaSalamanca, Spain; ^2^Department of Cell Biology and Pathology, University of SalamancaSalamanca, Spain; ^3^Department of Physiology and Pharmacology, University of SalamancaSalamanca, Spain; ^4^Centre for Interdisciplinary Research in Animal Health (CIISA), Faculty of Veterinary Medicine, University of LisbonLisbon, Portugal

**Keywords:** antidepressant treatment, early stress, fear conditioning, footshock, prepulse inhibition, sex differences

## Abstract

Early life stress is a major factor underlying the vulnerability to respond to stressful events later in life. The present study attempted to evaluate the role of prenatal stress affecting the development of stress-related disorders and their reversion by postnatal exposure to Sertraline (SERT), a front-line medication for medication for posttraumatic stress disorder (PTSD) in humans. To achieve this, adult male and female prenatally stressed (PS) or unstressed (Controls) offspring rats, following oral chronic treatment with SERT (5 mg/kg/day; from 1 month to 4 months old), or not, were studied prior to and after a traumatic event. First, anxiety-like behavior during the prepulse inhibition (PPI) test, a modulation of the startle reflex, was examined in all animals. Subsequently, the animals were subjected to a session of mild inescapable footshocks (IS; 0.35 mA, 5 s) in a shuttle box that was followed by 4 days of situational reminders in the aversive context. Prior to the footshocks no effects of PS or SERT were shown, and no changes in PPI and the habituation to the shuttle box were found. After them, PS led animals to exhibit behavioral alterations. When compared to the Controls, PS animals of both sexes displayed less rearing activity in the aversive environment. PS males responded less to footshock delivery and, in most of the animals, fear extinction was impaired. Moreover, the early postnatal exposure to SERT lessened the behavioral impact of PS in females, while in males it had no effect. Current results extend previous data from our laboratory, showing that PS heightened vulnerability to stress later on, and that SERT acts differently in males and females.

## Introduction

Stress response is described as a fundamental adaptation for an organism to cope with emergencies (McEwen, 2008). When this exceeds certain limits of intensity, the potentially beneficial alterations earlier mentioned can cause pathological states, or exacerbate latent or pre-existing morbid states (McEwen, 2008; Pereira-Figueiredo et al., 2015). In humans, the inability to adequately respond to a trauma or stressful situation may lead to psychopathological disorders, such as depression or posttraumatic stress disorder (PTSD; Morris et al., 2012; Careaga et al., 2016). Human symptoms of PTSD involve the development of long-lasting symptoms following the exposure to a life-threatening experience, which includes an exaggerated sensitization to fear, an increase in startle response, hypervigilance, helplessness and sensitization of the physiological responses to re-stress (Careaga et al., 2016). However, not all individuals that experience the same stressful trauma develop a disorder. To date, the definitive factors triggering the susceptibility to cope with stress have not yet been identified. Risk factors may include individual neurobiology as well as past experiences (Gunnar and Quevedo, 2008).

In recent decades, the incidence of reports indicating the higher vulnerability of children born of mothers that suffered stress during pregnancy to psychiatric disorders has raised new concerns regarding the effects of stress (Kajantie and Räikkönen, 2010; Lehrner et al., 2014). Early adverse experiences, including prenatal stress, have profound and long-lasting effects on the development of neurobiological systems that may “program” later vulnerability to stressful events (Gunnar and Quevedo, 2008; Kjær et al., 2010; Green et al., 2011; Xiong and Zhang, 2013).

In the past few years, several animal models of prenatal stress (PS) have been developed (Takahashi et al., 1990; Griffin et al., 2003; Koenig et al., 2005; Louvart et al., 2005; Abe et al., 2007). In rodents, PS has been linked to many changes in neurotransmitter systems, neuroendocrine function and the behavior of offspring (Kapoor et al., 2006; Wilson et al., 2013). Indeed, prenatally stressed animals tend to exhibit memory impairment (Kosten et al., 2006; Markham et al., 2010), develop higher emotional reactivity (Abe et al., 2007), higher levels of anxiety and fear (Griffin et al., 2003), and depression-like behaviors in adulthood (Salari et al., 2016). This type of early stress has also been shown to disturb noradrenergic and serotonergic homeostasis (Mastorci et al., 2009) and likely to predispose the offspring to the development of mood-related disorders later on in life.

Given the importance of the serotonergic system on anxiety and impulsiveness, including the modulatory effects of serotonin on stress responsiveness (Hashimoto et al., 1999; Kim et al., 2002; Goel et al., 2014; Wilson et al., 2014), in the present study we hypothesized that postnatal Sertraline (SERT) exposure is able to reverse the deleterious effects of early stress. SERT is a selective serotonin reuptake inhibitor (SSRI) used as the front-line medication for treating PTSD in humans (Davidson et al., 2001; Hien et al., 2015). Similar to other SSRIs (Salari et al., 2016), the ability of SERT to relieve PTSD- anxiety- or depression-like symptoms may depend on when the treatment is administered, its duration, and the sex of the individual. It has been suggested that the prolonged administration of SERT is needed to achieve clinical improvement (Kim et al., 2002). In fact, in human patients, treatments with SSRIs could last for years (O’Leary et al., 2013). Previously in our laboratory, treatment with a low dosage of SERT (5 mg/kg/day) for 8 days, in adult rats, was not enough to normalize all the deleterious effects of the stressful procedures (Pereira-Figueiredo et al., 2015). However, the same dosage, when given along adolescence, proved to be effective in reversing the impact of prenatal stress at adulthood (Pereira-Figueiredo et al., 2014). Therefore, in the present study, we hypothesized that the prolonged administration of SERT is needed (SERT given throughout 3 months, beginning at the first month of age), and more effective if given during early stages of development, restoring the behavioral impairments that prenatal stress may have induced.

The emergence of most of stress-related disorders is totally dependent on confrontation with a traumatic event (Kjær et al., 2010). With this in mind, in the present study, the behavioral outcomes of prenatal stress were assessed in adult offspring, following treatment with SERT or non-treatment; first, in a condition of pre-trauma, by using the prepulse inhibition (PPI) test and then, after being exposed to a stressful condition. The PPI, a modulation of the startle reflex, is a diagnostic tool believed to index essential mechanisms in the neural control of behavior (Geyer, 2006). In fact, although deficits in PPI were originally identified in schizophrenic patients (Koenig et al., 2005), reflecting a poor somatosensory integration, this condition has also been observed in several disorders related to anxiety and fear (Jensen et al., 2007; Ishii et al., 2010).

After being tested using the PPI, the animals were subjected to a contextual fear conditioning (CFC) paradigm where they were exposed to a mild session of inescapable shocks (IS) in a shuttle box, followed by situational reminders. This paradigm probes the acquisition of conditioned emotional responses and their extinction, when facing a given context, previously paired with some emotional stimuli (such as the footshocks). Induction of a stressful situation from which the animal cannot escape, has been previously described by several authors (Golub et al., 2009; Girardi et al., 2013; Hoffman et al., 2014) as a very useful tool to study the neurobiology of stress- related disorders, such as PTSD. Usually, PTSD patients exhibit an intense memory of a traumatic event, which resists extinction, and can persist for months, years or decades (Careaga et al., 2016).

Gender differences are often reported in humans, regarding disease prevalence and its symptoms (Seney and Sibille, 2014), showing that women are twice as likely as men to develop stress- and anxiety-related psychiatric disorders (for review see Maeng and Milad, 2015). Also, there are differences in the responses of men and women to antidepressant treatments (Sloan and Kornstein, 2003), sugesting that there are sex-specific differences in the behavioral response to stress and antidepressants (Leuner et al., 2004; Duchesne et al., 2009; Simpson and Kelly, 2012). Hence, we anticipated that the inclusion of both male and female animals in our study might enhance the validity of the SERT treatment, reversing stress-induced changes.

In the present study, our main goal was to address the effects of maternal stress on the susceptibility of offspring to stress-related diseases, and to evaluate to what extent these consequences could be reversed by postnatal exposure to SERT in animals of both sexes. Furthermore, the understanding of the mechanisms underlying the hypothetical sex-differences in the susceptibility to stress may provide insight into the etiology of these disorders and predict treatment outcomes.

## Experimental Procedures

### Animals

Virgin female Wistar rats (*n* = 14) weighing 250 g were obtained from outbred rats from our own animal facility at the University of Salamanca. Vaginal smears were collected daily for 8 days before mating to determine the stage of the estrous cycle and the day of conception. On the day of proestrus, sexually experienced male Wistar rats (1:1) were introduced for mating. For all experiments, animals were allowed *ad libitum* access to food and water, and maintained on a regular light-dark cycle (lights on: 07:00 am–19:00 pm) with a constant temperature of 21°C. The animals were handled and cared for according to the guidelines of the European Communities Council Directive (2010/63/CE) for the care and use of laboratory animals. This research was carried out under the supervision of the Animal Care and Use Committee of the University of Salamanca, with the approval number #125541368712-25-02-2012.

### Prenatal Stress Exposure

**Figure 1:**
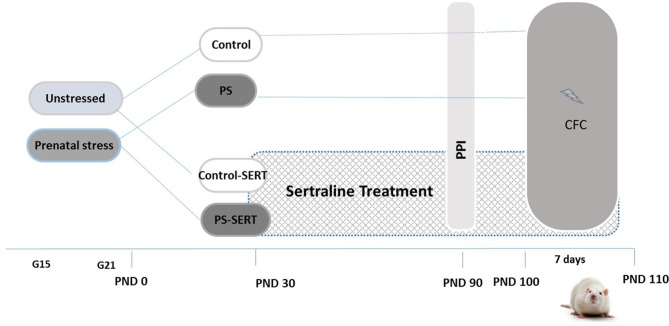
Experimental design and timeline. Non-stressed or prenatally stressed offspring of both sexes, treated with Sertraline (SERT) (Control-SERT and PS-SERT; 5 mg/kg/day), or not (PS and Control; *n* = 14 animals on each group and sex, respectively), were exposed to the prepulse inhibition (PPI) test at postnatal day 90 (PND 90), and to the Contextual fear conditioning paradigm (CFC; starting at PND 100). Abbreviations: G15 and G21, gestational days 15 and 21.

As previously described (Pereira-Figueiredo et al., 2014), pregnant female rats were randomly assigned to either the stressed or non-stressed groups (*N* = 7 per group) and individually housed in plastic breeding cages (Figure 1). Stress consisted of placing the females, in the last week of gestation (days 15–21), into transparent cylinder restrainers (7 cm diameter, 19 cm long), under a bright light (50 W bulb) directed onto the surface of the restrainer for 45 min three times per day (at 09:00 am, 12:00 and 16:00 pm). Control mothers were left undisturbed, and handled only during routine activities (cleaning, etc.). All stressed and control mothers gave birth naturally, and no differences in litter sizes, in offspring male-to-female ratio or in pre-weaning-mortality were found. Offspring were weaned at 21 days of age and were separated into group cages housing four animals of the same sex and treatment, with the criterion that groups included no more than two pups from the same litter.

### Drug Administration

Starting at postnatal day 30 (PND 30) and continuing for 90 days until the end of experiments (PND 120; Figure 1), pups from each condition, Control or PS, were subdivided for receiving either treatment with SERT (Control-SERT and PS-SERT; *N* = 9 per sex and group), or filtered water (Control, PS; *N* = 14 per sex and group). Based on previous reports (Pereira-Figueiredo et al., 2014, 2015), SERT was given orally (Besitran© Pfizer S.A. Madrid, Spain) at a dose of 5.0 mg/kg/day. Using calibrated bottles, which were monitored every 2 days, liquid consumption was controlled, and the dose of the drug was adjusted on the basis of the liquid consumed and mean body weight. During this period, the rats were kept in groups of four animals in polycarbonate boxes (45 × 30 × 20 cm), with unrestricted access to food.

### Prepulse Inhibition (PPI)

At PND 90, all animals of both sexes were tested using PPI, in a simple paradigm routinely implemented in our laboratory (Castellano et al., 2009) using the SR-LAB system (SDI, San Diego, CA, USA). Before testing, the rats were habituated to the experimental conditions, especially regarding their placement into the apparatus. The acoustic startle reflexes (ASR) were measured in six identical startle response systems simultaneously. Acoustic stimulus intensities and response sensitivities were calibrated (using an SR-LAB Startle Calibration System) to be nearly identical in each of the six SR-LAB systems (maximum variability <1% of stimulus range and <5% of response ranges). The background noise of 65 dB SPL was generated throughout the entire session in order to avoid interference from external noise and ensure equal experimental conditions. A session consisted of an acclimatization period of 5 min and 64 trials presented pseudo-randomly. Sixteen of the trials were a single noise pulse (115 dB, 20 ms of bursts), used to determine the ASR. The remaining 48 trials were a white noise prepulse presented at different intensity levels (65, 70, or 80 dB SPL) of 20 ms-duration, followed by a startling stimulus with an interstimulus interval of 50 ms. Whole-body movements corresponding to the startling responses were recorded with a piezoelectric accelerometer and converted into analogical signals that provided the latencies and amplitudes of the ASR. The percentage of the magnitude of PPI was calculated for each respective prepulse intensity according to the following formula: % PPI = 100 − (100 × startle amplitude in prepulse followed by pulse trial)/(startle amplitude on pulse trial alone). The PPI latency was the time taken between each prepulse intensity stimulus and the corresponding response, expressed in milliseconds. This test was performed between 10:00 am and 14:00 pm in a separate room away from the other behavioral test. Immediately after the test was concluded, all animals were weighted and the females were subjected to estrous cycle determination.

### Estrous Cycle Determinations

Vaginal smears were obtained by dipping a sterile swab (0.6 mm diameter, 0.025 in Fischer Scientific) in sterile saline, and then gently swabbing the vaginal lumen. The swabs were smeared onto labeled glass slides that were previously cleaned with 95% ethanol. The cells were fixed with 95% ethanol for 15 min and then air-dried before staining with hematoxylin-eosin. The vaginal smears were examined for estrous cycle assessement using an Olympus Microscope (40×). Following previous criteria, proestrus, estrous, metestrus and diestrus phases were identified in a 4–5 day-cycle (Marcondes et al., 2002). Rats in proestrus had the highest serum estrogen levels (with a high number of nucleated epithelial cells in vaginal smear), while by contrast, rats in estrus presented the lowest estrogen levels (with anucleated cornified cells). In metestrus, rats’ vaginal smear depicted a high number of white blood cells (WBC) and nucleated cornified cells. In diestrus, besides the predominance of WBC in the vaginal smear, some epithelial cells were also present.

### Contextual Fear Conditioning Test

Seven days after the PPI procedure ended (at PND 100), the animals were subjected to the CFC paradigm. The shuttle box (25 cm depth × 29 cm height × 25 cm width) with an anterior transparent wall (Letica Scientific Instruments, Spain) was used as the conditioned stimulus (CS); footshocks were used as the unconditioned stimulus (US). This test consisted on 7 day consecutive sessions lasting for 3 min. The first 2 days allowed the animals to become habituated to the experimental conditions—the animals were introduced into the apparatus and left undisturbed. On the third day, animals underwent fear conditioning and, after 1 min of acclimatization, a sequence of three mild shocks (5 s, 0.35 mA, with 20 s between each one) were administered through a metal grid floor, with no warning light signal either before or during each shock. Then, the rats were allowed to stand for another minute before returning to their home cages. Shock sensitivity was assessed by the occurrence of jump reactions and audible vocalizations (Jourdan et al., 1995; Kosten et al., 2006). In order to reduce the inherent effects of differences in the estrous cycle, only females that were not in proestrus were used on the day the shocks were administered (Marcondes et al., 2002). For extinction training, the animals were re-exposed during the four remaining daily sessions, to the testing apparatus. These sessions occurred without shocks, and served to introduce a situational reminder of the shocks (Markham et al., 2010).

Behavior was videotaped using a camera (Sony HDR-CX 220) mounted on the ceiling and was later scored by observers, blind to the experimental conditions. The unconditioned/acquisition of fear was given by the percentage of time the animals spent motionless when exposed to the context—*freezing*: at least 3 s of no detectable movements (only *vibrissae*); or *crouching* (similar to *freezing*, but with detectable movements of the neck). Both postures were analyzed at the same time and defined as *defensive* (DEF; Yang et al., 2004). The extinction of fear was defined as being acquired when less than 10% of the time was DEF at four sessions after the shocks. Also, the number of *rearings* (vertical activity) was recorded during the entire procedure.

### Statistics

Statistical analyses were performed using IBM^®^ SPSS^®^ software, version 20 (IBM Crp. and SPSS Inc., Chicago, IL, USA, 2011). Differences between groups were analyzed by ANOVA (one, two and three way), followed by the Fisher-PLSD-test for *post hoc* comparison, if necessary, and ANOVA mixed (or “SPLIT-PLOT”) with the Bonferroni-test. Mean differences were subjected to pairwise comparison by Student’s *t*-test, using the Levene Test for equality of variances. Pearson’s coefficient was used to determine correlations. To test whether the animals reached “fear extinction” criterion or not, chi square analyses were used. Differences were regarded as statistically significant when *p* < 0.05.

## Results

### PPI Measures

**Figure 2:**
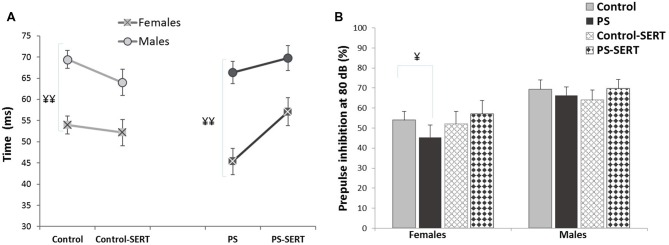
PPI latency **(A)** and PPI values **(B)**, obtained in the animals of both sexes at the age of 90 days, previously submitted or not to prenatal stress (PS and Control) and treated with SERT (5 mg/kg/day; PS-SERT and Control-SERT). *n* = 9–14 animals per group and sex (Mean values ± SEM). ^¥¥^*p* < 0.01 and ^¥^*p* < 0.05 indicate a main effect of sex.

To evaluate the behavioral effects of the administration of PS and SERT, the analyses were performed using the highest prepulse intensity level (80 dB SPL), as the highest inhibitions using PPI were obtained with the highest intensities. The 2-way ANOVA (with group vs. sex as factors) showed no significant effects of the treatments with respect to the PPI levels (*F*_(3,71)_ = 0.91, n.s.), although, a sex effect was found (*F*_(1,71)_ = 14.2, *p* < 0.001). As shown in Figures 2A,B, males exhibited higher PPI values and longer latency than females. The only significant differences were detected among animals that were not taking SERT (*p* < 0.001), regardless of the previous stress applied. Moreover, in females, no correlations were found between PPI values and the phase of the estrous cycle (*r* = −0.021).

### Contextual Fear Conditioning

#### Habituation to Context

During the habituation sessions, prior to the IS, the animals of all groups showed little defensive behavior, and the percentage of time spent DEF was comparable for all of the animals (mixed 3-way ANOVA with group, sex and sessions as factors, *F*_(7,98)_ = 1.92, n.s., Figures 4A,B).

**Figure 3:**
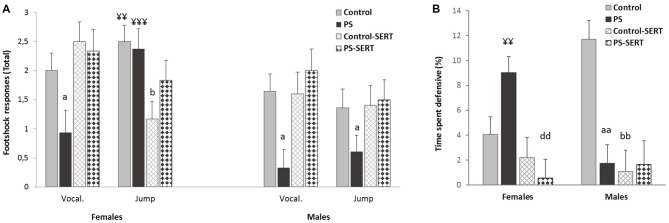
Effects of prenatal stress and SERT treatment (5 mg/kg/day) over the course of the context-shocks pairing session. **(A)** Vocalization and jump intensity responses to footshock delivery, in the animals of both sexes; **(B)** time that rats spent defensive (expressed in percentage). Each bar represents the sum of the response’s intensity (ranging from 0 to 1), to the three footshocks presentation (of 0.35 mA each), expressed as Means + SEM (*n* = 8–9 animals per group and sex). ^aa^*p* < 0.01 and ^a^*p* < 0.05, indicate the effect of prenatal stress; ^b^*p* < 0.05 and ^bb^*p* < 0.01 indicate the effect of SERT in controls; ^dd^*p* < 0.01 indicate the effect of SERT in PS females; ^¥¥^*p* < 0.01; ^¥¥¥^*p* < 0.001, indicate the effect of sex within non-treated animals.

**Figure 4:**
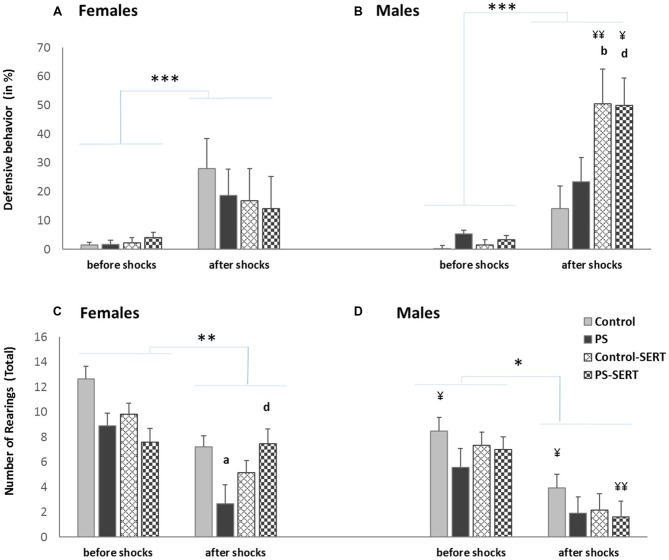
Effects of a session of inescapable shocks (IS) in the time the animals of both sexes spent displaying defensive behavior (in%) **(A,B)**; and in the number of rearings **(C,D)** during the CFC paradigm test. Before shocks: each bar represents the mean values + SE in the two sessions before the IS; after shocks: each bar represents the mean values + SE in the three sessions after the IS. ^a^*p* < 0.05, indicates the differences as effect of prenatal stress; ^b^*p* < 0.05, indicates the differences as effect of SERT in control animals; ^d^*p* < 0.05 indicates the significant effects of SERT in prenatally stressed females; ^¥¥^*p* < 0.01 and ^¥^*p* < 0.05, indicate the differences between sexes; ***p < 0.001, **p < 0.01 and *p < 0.05 indicate the significant change between the sessions before vs. after IS.

#### Context-Shocks Pairing

The sensitivity to IS was assessed by the frequency of responses such as jumps and vocalizations. The 2-way ANOVA revealed the effect of early stress affecting the number and intensity of vocalizations (*F*_(1,49)_ = 7.38, *p* < 0.001, Figure 3A). PS animals of both sexes vocalized less upon their reaction to shocks than the control group, and in both sexes SERT acted by reversing it. Regarding the jump reaction, the ANOVA revealed differences between experimental groups (*F*_(1,49)_ = 9.8, *p* = 0.003) with group * sex interaction (*F*_(1,49)_ = 9.8, *p* = 0.003). Within non-treated animals, the females responded more intensely than males (*p* < 0.01). In males, early stress affected this measure and PS males jumped significantly less intensely than the controls in response to footshocks (Figure 3A).

During the IS session, fear conditioning was observed. DEF behaviors increased significantly, when compared to the previous sessions (3-way ANOVA with group, sex and session as factors) *F*_(7,98)_ = 35.13, *p* < 0.001, revealing the effect of the previous treatments (*F*_(7,98)_ = 7.8, *p* = 0.002). *Post hoc* analyses showed that for both sexes, the animals taking SERT spent less time *crouching* or *freezing* than their control counterparts (*p* < 0.001, Figure 3B). Moreover, within early stressed animals there were differences between sexes, and PS females displayed more time exhibiting DEF responses than males.

#### Contextual Fear Extinction

Re-exposure to the conditioning box, in the absence of shocks, elicited defensive behaviors, in all animals (*F*_(7,98)_ = 65.6, *p* < 0.001, Figures 4A,B). After the shocks, the 2-way ANOVA revealed differences regarding fear demonstration towards the box, as an effect of group (*F*_(1,49)_ = 12.13, *p* < 0.001) and sex (*F*_(1,49)_ = 4.76, *p* = 0.034) with group * sex interaction (*F*_(1,49)_ = 4.48, *p* = 0.007). *Post hoc* comparisons indicated that, in males, SERT treament increased the time spent defensive, regardless of the previous stress condition (*F*_(3,49)_ = 18.4, *p* < 0.001, Figure 4B). Within the treated animals, the differences reached significance when males were compared with females (*F*_(1,49)_ = 9.8, *p* < 0.01).

**Figure 5:**
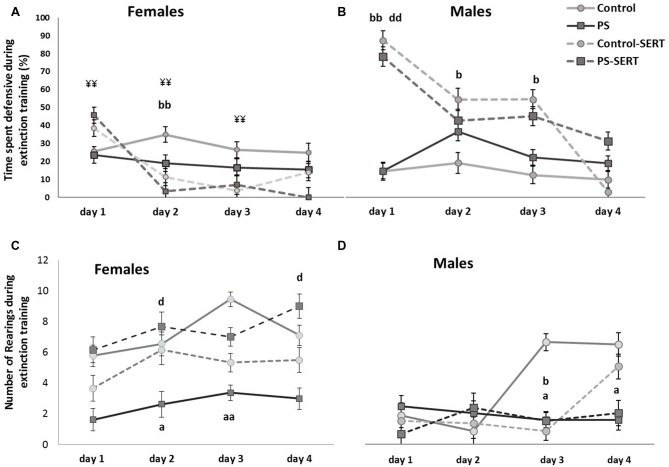
Effects of prenatal stress and SERT treatment (5 mg/kg/day) in the time, the animals of both sexes, spent displaying defensive behavior **(A,B)**; and in the number of rearings **(C,D)**, when they were re-exposed to the shuttle box, after the footshocks delivery, i.e., during extinction training (*n* = 8–9 animals per group and sex). ^bb^*p* < 0.01 and ^b^*p* < 0.05, indicate the significant effect of SERT in control animals; ^d^*p* < 0.05, and ^dd^*p* < 0.01, indicate the significant effects of SERT in PS animals; ^¥¥^*p* < 0.01, indicate the differences between sexes in the animals taking SERT (Mean values ± SEM).

To explore the effects of the drug and prenatal stress, on fear extinction training, sessions were repeated daily and the analyses were performed separately from day 1 to day 4 post-shocks (Figure 5). In the 1st day in the absence of shocks, *post hoc* analyses showed that SERT treatment increased the demonstration of fear towards the box (*F*_(3,49)_ = 15.4, *p* < 0.001, Figures 5A,B). In males, the effect of SERT reached significance and continued to be significant during the 2nd and the 3rd days of extinction training (*F*_(3,49)_ = 6.1, *p* < 0.01, *F*_(3,49)_ = 8.4, *p* < 0.01, Figure 5B). Within non-treated animals of both sexes, DEF behaviors did not significantly change, as well as previous stress did not influence fear demonstration.

On the 4th day after the shocks were administered, the 2-way ANOVA no longer revealed differences between groups, or sexes (*F*_(1,49)_ = 1.2, n.s. in females and *F*_(1,49)_ = 1.5, n.s., in males). However, at this session, when each experimental group was compared, differences were found in the number of animals that managed to reach the fear extinction criterion (less than 10% of time DEF; Table 1). Overall, SERT did help the animals in the extinction of fear from the context (*X*^2^ = 7.99, df = 7, *p* = 0.046), with the only exception being that of the PS males, where SERT had no apparent effect. In males, early stress affected the fear extinction, and differences were revealed when the PS males were compared to the controls (*X*^2^ = 8.2, df = 3, *p* = 0.041). In females, SERT was quite effective and helped the animals to manage the extinction of fear (*X*^2^ = 5.98, df = 3, *p* = 0.024 and *X*^2^ = 6.22, df = 3, *p* = 0.012, in controls and PS females, respectively).

**Table 1:** Percentage of animals that managed to extinguish fear of the box.

Experimental group	Females	Males
Control	55.5 ± 8.7	85.7 ± 5.7
Control-SERT	83.3 ± 7.1^b^	100.0 ± 0.0
PS	50.0 ± 8.2	44.4 ± 8.6^a^
PS-SERT	100.0 ± 0.0^d^	66.6 ± 7.6

*Percentage of animals that exhibited less than 10% of time defensive in the 4th session after footshocks, in the different experimental groups exposed to the contextual fear conditioning (CFC) paradigm. ^a^p < 0.05, indicates the differences as effect of prenatal stress; ^b^p < 0.05, and ^d^p < 0.05, indicate the significant differences as an effect of SERT in the controls and prenatally stressed (PS) females, respectively*.

#### Rearings

When the experimental groups were compared, before the shocks, the ANOVA revealed no differences in the number of rearings (*F*_(1,49)_ = 14.1, n.s., Figures 4C,D). After the exposure to the shocks, the exploratory activity significantly decreased in all animals (3-way ANOVA, *F*_(3,49)_ = 55.1, *p* < 0.001).

During the days following the IS, most animals recovered the exploratory activity, while the fear of the box decreased (*r* = −0.41**), except for the PS animals (Figures 5C,D). As a consequence, during extinction training, the differences revealed by ANOVA became evident, for both sexes (*F*_(3,49)_ = 3.8, *p* = 0.016; *F*_(3,49)_ = 3.2, *p* = 0.031, in females and males, respectivelly). *Post hoc* analysis showed the effect of prenatal stress affecting the number of rearings, where the PS animals of both sexes displayed less exploratory activity than the control animals, an effect that reached significance in females (*p* = 0.028, Figure 4C). Moreover, SERT treatment reversed the effects of the shocks in females PS, but this did not occur in PS males.

## Discussion

During the past few decades, it has been shown that early life stressors, such as prenatal stress, can alter the threshold of vulnerability of the brain systems to subsequent stressors (Abe et al., 2007; Kajantie and Räikkönen, 2010). However, some behaviors, even if apparently changed by the PS procedure, are not displayed, when measured under low anxiety conditions (Mastorci et al., 2009; Kjær et al., 2010; Green et al., 2011). The main findings of the present study reinforce this idea, where previous to exposure to a session of IS any observable behavioral differences were detected as an effect of early stress. Although, after the IS, PS animals exhibited clear signs of disturbance when faced with new challenges.

Once the shocks had been administered, the PS animals of both sexes exhibited difficulties in adapting to the aversive context and to re-stress. When compared to the controls, they displayed less number of rearings, a clear sign of the lack of motivation. Moreover, the PS males were less responsive to footshock delivery and most of them did not manage to display fear extinction. These results are in line with what happens in humans with PTSD, where the symptoms of the disease are exacerbated or only revealed in the presence of stressful stimuli (Careaga et al., 2016). The factors affecting the differences in the susceptibility to stress-related disorders and its reversion, depending on the individual’s sex, are still not clear (Seney and Sibille, 2014; Maeng and Milad, 2015). Our data show that the consumption of SERT, starting at the beginning of the adolescence through adulthood, was effective at remediating the effects of prenatal stress, although more efficiently in females than in males. Most importantly, the present study shows the clear differences in the effectiveness of SERT, based on sex, which only became evident after exposure to footshocks.

### Previous to Is Neither Prenatal Stress nor SERT Induced Behavioral Changes

Prenatal stress may disturb the dopaminergic mesolimbic system, which is closely related to PPI modulation (Koenig et al., 2005; Geyer, 2006). Koenig et al. (2005) reported that the PPI of the acoustic startle response was diminished in PS adult male rats. Nevertheless, in the present study, we did not observe a clear relationship between prenatal stress and PPI values. Also, before the IS, when the animals were exposed to the shuttle box, an innocuous/unfamiliar environment (Girardi et al., 2013), neither prenatal stress nor SERT affected the unconditioned fear or the rearing activity. According to Lever et al. (2006), rearing is an expectable behavior in rodents in their response to novelty, as it promotes learning about the spatial environment. However, rearing also represents the motivation to escape, as it is often focused on the boundaries of an enclosure, suggesting this is highly modulated by the motivation to explore, and thus, by fear and anxiety.

Using the same paradigm, we have previously reported that under basal conditions, neither prenatal stress nor SERT treatment induced remarkable alterations in behavior and had no effects on the mean startle reflex values (Pereira-Figueiredo et al., 2014). Other authors have also reported inconsistent results regarding the effects of this type of early stress (Kjær et al., 2010), or failed to demonstrate changes on PPI modulation (Jensen et al., 2007), or anxiety-/depression-like behaviors (Salari et al., 2016), when using other SSRIs under basal conditions. In addition, our data show that there are differences in PPI and latency values between males and females. The reported sex-differences could be due to changes in reproductive hormones, in particular estrogen (E_2_) (Swerdlow et al., 1997). However, in accordance with Adams et al. (2008), and a previous work from our laboratory (Pereira-Figueiredo et al., 2015), we did not find any correlation between E_2_ levels and PPI, which were tested in random phases of the estrous cycle.

### Prenatal Stress Affected the Response to Footshock

With the administration of footshocks all animals responded with vocalizations or jumps and/or *freezing*. These types of reactions are considered important defensive reflexes that may help to protect the animal from injury (Jourdan et al., 1995; Hillman et al., 2005; Popa et al., 2008). The present data show differences in their responses to footshocks according to sex and treatment. Whereas control females were consistently more reactive than males (jumped significantly more), the males froze more often, a finding that is in agreement with the literature (Blanchard et al., 1991; Kosten et al., 2006), while PS males became unresponsive. Our data show that early stress affected the response to footshocks in males, where PS males jumped, vocalized and froze less when exposed to the shocks, in comparison with the controls and with their female counterparts.

It has been suggested that the nociceptive system of offspring may be altered by exposure to early stressors. According to previous authors (Takahashi et al., 1990), the opioid endogenous changes during a stressful pregnancy can significantly affect the mediation of nociceptive systems in the offspring that may be different in each sex. However, recently, Wilson et al. (2013) reported that prenatal stress did not induce any alterations in the nociception of PS rats. The reduced reactivity to painful shocks observed in PS males may not be due to a lower sensitivity to shocks but, as suggested by Leuner et al. (2004), governed by other patterns of emotional behavior, probably reflecting differences in the activation threshold in some brain structure.

### The Effects of Prenatal Stress in Fear Extinction

On each time animals were re-exposed to the box, after the shocks, a fear response was elicited and they displayed an overall suppression of activity. The animals had learned, by contextual conditioning (Yang et al., 2004), that the box was a hostile environment and responded by *freezing* or *crouching*, postures requiring the animal to be motionless but alert (Blanchard et al., 1991; Popa et al., 2008). In nature, these are the normal defensive behaviors that rodent animals adopt in preparation for a possible coup or attack (Blanchard et al., 1991; Hashimoto et al., 1999; Yang et al., 2004). Being alert is a protective feature of all organisms, but at the same time withstanding it, is also detrimental to the organism (Burghardt et al., 2013), suggesting the importance of the feature of fear extinction.

For many years, *freezing* was considered an index of anxiety (Hashimoto et al., 1999). Nevertheless, rats with a mild level of fear, associated with a given context, may not freeze but limit their locomotor activity and adopt the *crouching* behavior (Popa et al., 2008). Such assessment is very informative because of its sensitivity to lower levels of fear (Blanchard et al., 1991). Whichever the case, the formation of conditioned fear and its extinction are dependent on learning processes that depend on the hippocampal receptors, the medial prefrontal cortex (Maeng and Milad, 2015) and the amygdale (Markham et al., 2010). Therefore, it could be expected that PS animals might have some disruption in the acquisition and extinction of fear (Griffin et al., 2003; Green et al., 2011).

In fact, our data indicate that early stress affected behaviors during the extinction training. In all animals, a lasting reduction of fear was replaced by the behaviors adopted before the shocks, except in PS animals. Our data show that PS animals adopted relatively to the environment, a passive strategy, displaying a lack of responses, neither exploring nor taking a defensive behavior, clear signs of the lack of motivation (Hillman et al., 2005; Lever et al., 2006). Moreover, we found that while control animals gradually re-explored the experimental box, the PS animals of both sexes did not resume this activity, as they were unable to get used to the context that was no longer aversive. Also, Louvart et al. (2005) observed that after being submitted to footshocks, their PS females exhibited a decrease in the exploratory activity and also in their ability to manage to extinguish fear when compared to controls. Previously, Wilson et al. (2013) similarly found that PS impaired fear extinction, in rats of both sexes. Besides, Markham et al. (2010) reported that the extinction of the memory of conditioned fear in PS animals depended on the sex, being disrupted in male but not in female rats. Accordingly, our data indicate that, after 4 days of extinction training, only a small number of PS males acquired fear extinction.

### The Effects of SERT on Fear Conditioning and its Extinction

In the present study, we suggest that SERT helped the animals to become faster accustomed to stress conditions. Importantly, our data indicated that SERT accelerated the extinction of fear in all animals, reversed the apparent stress-induced lack of motivation, and recovered exploratory activity, except in PS males. According to previous works (Davidson et al., 2001; Kim et al., 2002; Wilson et al., 2014), it might be expected that SERT could be able to lessen the impact of a traumatic event in rodents.

As far as we know, few studies have examined the effects of antidepressants specifically in the extinction of aversive memories, and in these studies, differences were reported in their effects (Melo et al., 2012; Burghardt et al., 2013; Wilson et al., 2014). Wilson et al. (2014) reported that in male rodents, despite signs of attenuating PTSD, SERT did not diminish anxiety and even increased the noradrenergic response. In agreement, our data show that during the extinction process, SERT elicited a significant increase in the amount of time spent defensive that was followed by a marked decline throughout all of the sessions, except for males submitted to PS. Apparently, the treatment with SERT was quite effective in most of the animals, reversing the IS-induced behavioral impairments, even though, it had no effect in PS males.

According to the literature, the administration of antidepressants should normalize the activity of the hypothalamic-pituitary-adrenal axis (Leuner et al., 2004), and control the sympathetic drive (Wilson et al., 2014). However, women are often more responsive to antidepressants than men (Sloan and Kornstein, 2003). Accordingly, we have recently reported that in rodents, SERT was more effective in females than in males (Pereira-Figueiredo et al., 2015). Indeed, differences in 5-HT levels, related to sex of naïve animals, have already been reported in many structures of the corticolimbic region known to regulate stress and emotion processing (Duchesne et al., 2009; Goel et al., 2014). The spontaneous firing rate of dorsal raphe neurons (DRN) in males is more than 40% higher than in females (Duchesne et al., 2009). Furthermore, quite recently Goel et al. (2014) reported there are sex-differences in the cellular activation of the 5-HT1A receptors in the DR, in response to stress. The present data suggests that these differences are heightened when the animals suffer from an early stress, and could be related to the differences we found between males and females in response to SERT treatment.

In any event, differences based on sex causing vulnerability to developmental challenges and the associated treatments to prevent their outcome must be taken into account in further studies.

## Conclusion

The present study supports the hypothesis that events in the womb are a putative biological risk for the development of stress-related disorders. Our data demonstrate that prenatal stress causes animals to become more vulnerable to stress in adulthood, and seem to indicate that the emergence of behavioral impairments requires the exposure to a traumatic or stressful event. In fact, the effects of early stress are manifested only if the animals are confronted with a mild session of IS. Surprisingly, we also found that the exposure to footshocks gave rise to differences dependent on sex in SERT effectiveness. The postnatal treatment with SERT was able to reverse the behavioral impairments stress-induced more efficiently in females than in males. We hope the present study helps to elucidate the mechanisms and treatments needed to be followed in conditions of uncontrolled fear or stress disorders in humans, highlighting the differences between sexes.

## Author Contributions

The authors IP-F, DEL, CS and ASR cooperated in the design of the study. The authors IP-F, OC, CS and GF-D carried out the laboratory procedures and contributed to the interpretation of data. All authors contributed to the editing of the manuscript and have approved the final manuscript.

## Conflict of Interest Statement

The authors declare that the research was conducted in the absence of any commercial or financial relationships that could be construed as a potential conflict of interest.
